# Diagnostic accuracy of macular ganglion cell-inner plexiform layer thickness for glaucoma detection in a population-based study: Comparison with optic nerve head imaging parameters

**DOI:** 10.1371/journal.pone.0199134

**Published:** 2018-06-26

**Authors:** Victor Koh, Yih-Chung Tham, Carol Y. Cheung, Baskaran Mani, Tien Yin Wong, Tin Aung, Ching-Yu Cheng

**Affiliations:** 1 Singapore Eye Research Institute, Singapore National Eye Centre, Singapore; 2 Department of Ophthalmology, National University Health System, Singapore; 3 Department of Ophthalmology and Visual Sciences, The Chinese University of Hong Kong, Hong Kong, China; 4 Duke-NUS Graduate Medical School Singapore, Singapore; 5 Yong Loo Lin School of Medicine, National University of Singapore; University of California San Diego, UNITED STATES

## Abstract

**Aims:**

To determine the diagnostic performance of macular ganglion cell-inner plexiform layer (GCIPL) thickness measured by spectral-domain optical coherence tomography (SD-OCT) for glaucoma detection in a Chinese population in comparison with optic nerve head (ONH) and retinal nerve fiber layer (RNFL) parameters measured by both SD-OCT and Heidelberg Retina Tomography 3 (HRT-3).

**Methods:**

Adults aged 40 to 80 years were recruited from the population-based study (n = 3353, response rate 72.8%). Macular cube 200x200 scan was performed with Cirrus SD-OCT (version 6.0, Carl Zeiss Meditec Inc, Dublin, CA) for GCIPL thickness measurement. ONH and RNFL imaging was performed with Cirrus SD-OCT and HRT-3 (Heidelberg Engineering, Heidelberg, Germany). Glaucoma was defined according to International Society for Geographical and Epidemiological Ophthalmology criteria.

**Results:**

In total, 86 eyes of 60 subjects with glaucoma and 1709 eyes of 1001 non-glaucoma participants were included. The best performing parameters for Cirrus SD-OCT GCIPL, Cirrus SD-OCT ONH and HRT-3 were minimum GCIPL thickness (Area under receiver-operating curve [AUC] = 0.89, 95% CI 0.83–0.95), vertical cup-disc ratio (CDR) (AUC = 0.94, 0.91–0.98) and vertical CDR (AUC = 0.86, 0.81–0.92), respectively. At 85% specificity, vertical CDR measured using Cirrus OCT ONH scan showed the highest sensitivity (88.64%, 95% CI 75.4–96.2) compared to minimum GCIPL thickness with sensitivity of 60.53% (95% CI 46.4–73.0) (p<0.001). Inferior RNFL thickness (AUC = 0.84, 95% CI 0.91–0.97) measured by Cirrus SD-OCT was also superior to Cirrus SD-OCT GCIPL (p<0.007).

**Conclusions:**

The diagnostic performance of macular GCIPL scan is inferior compared to vertical CDR measured by Cirrus OCT ONH scan. Cirrus OCT ONH scan showed the best ability in detecting glaucoma in a Chinese population, suggesting it could be a good glaucoma screening tool in an Asian population.

## Introduction

Glaucoma is a major cause of irreversible blindness in the world and is estimated to affect more than 100 million people by the year 2040.[1] If diagnosed early, effective treatment can be implemented to retard visual loss, making glaucoma an important disease to screen. Studies have shown that structural changes of the optic nerve head (ONH) and retinal nerve fiber layer (RNFL) often precede the development of visual field defects.[2, 3] There are now several imaging modalities for the ONH and RNFL including confocal scanning laser ophthalmoscopy (CSLO), scanning laser polarimetry and spectral-domain optical coherence tomography (SD-OCT). These produce highly reproducible, objective and quantitative measurements of the ONH and RNFL.[4, 5]

Previous studies reported that macular ganglion cell layer measurements by SD-OCT have good diagnostic accuracy for detecting glaucoma when combined with visual field testing in the clinic setting.[6, 7] Retinal ganglion cell loss is one of the first layers within the retina to be affected by early glaucoma.[8] As such, the ability to image the macula, which comprises the region with thickest ganglion cell layer could be a promising screening tool for glaucoma in a population-setting. The Cirrus OCT macular cube scan is able to perform auto-segmentation of the retina and isolate just the ganglion cell and inner-plexiform layer (GCIPL) with good reproducibility.[9, 10] This measurement excludes the RNFL layer and as the ganglion cell layer thickness showed less variability compared to RNFL in normal population, hence including the RNFL in the ganglion cell layer analysis may affect the sensitivity in the detection of early glaucoma.[10] It has been shown that GCIPL measurement showed higher diagnostic ability than RNFL thickness in early glaucoma and similar diagnostic ability for moderate and severe glaucoma.[11, 12]

Currently, there is a lack of population-based studies to evaluate the diagnostic performance of macular GCIPL thickness for glaucoma detection in a population setting. Of note, compared to other ethnic groups, the diagnostic performance of glaucoma imaging tools in Asian population has been shown to be significantly lower. The aim of our study was to determine the diagnostic accuracy of macular GCIPL thickness in comparison with ONH and RNFL measures by both OCT and CSLO for glaucoma detection in a population setting.

## Materials and methods

This study comprised participants from the Singapore Chinese Eye Study (SCES), a population-based study of Chinese adults in Singapore, aged between 40 and 80 years. The details of SCES had been reported in detail elsewhere.[13] All the participants signed a written informed consent. This study adhered to the Declaration of Helsinki, and ethics committee approval was obtained from the Singapore Eye Research Institute Institutional Review Board.

### Study population

Participants were consecutively recruited between Feb 2009 and July 2010. All subjects underwent a full ophthalmic examination including measurement of visual acuity, subjective refraction, intraocular pressure (IOP), gonioscopy, and dilated fundus examination. We excluded those with macular disease, previous vitreo-retinal or refractive surgery and neurological diseases. Glaucoma was defined as having optic nerve features of glaucoma and RNFL defects found on fundus examination and corresponding visual field defects (as described below). This was based on the ISGEO (International Society of Geographical and Epidemiological Ophthalmology) criteria (Category 1 diagnosis for cross sectional prevalence surveys) which included features of glaucomatous optic neuropathy and corresponding visual field. [14]. The control group had intraocular pressure of < 21 mm Hg with open angles, healthy optic discs on clinical examination and normal visual fields (defined as mean deviation and pattern standard deviation within 95% confidence limits, and Glaucoma Hemifield Test within normal limits).

### Ocular examination and visual field tests

All patients underwent a standardized and complete ophthalmic examination at the Singapore Eye Research Institute. Subjective refraction and distance best- corrected visual acuity in Log MAR scores were measured by trained optometrists. Both the anterior and posterior segment examination was performed at the slit-lamp (Haag-Streit model BQ-900; Haag-Streit, Switzerland) using a 78 Diopter lens, which included measurements of vertical diameters of the optic disc and cup. All the eyes had the IOP measured using the Goldmann applanation tonometer (Haag-Streit, Switzerland). Keratometry and axial length measurements were obtained from the IOL master. We performed visual field testing using static automated white-on-white threshold perimetry (Swedish interactive threshold algorithm fast 24–2, Humphrey Field Analyzer II; Carl Zeiss Meditec, Inc.). Glaucomatous optic neuropathy had the following characteristic features including localised rim notching or thinning, RNFL defects or disc hemorrhage, and/or disc asymmetry between the 2 eyes. A visual field was defined as reliable if the fixation losses were less than 20%, false-positive rates and false-negative rates were less than 33% each. A visual field would be consistent with glaucoma if the following criteria is met: the presence of three or more significant non-edge contiguous points (p<0.05) and at least one point with p<0.01 in the pattern deviation plot, and Glaucoma Hemifield Test is “outside normal limits”.

### Imaging modalities

All the 3 imaging scan types including spectral-domain OCT macular GCIPL scan, Cirrus SD-OCT ONH scan and CLSO scan, were performed in the same visit for each study eye after pupil dilation using tropicamide 1% and phenylephrine hydrochloride 2.5%. Only good quality images were included for analysis.

Spectral-domain OCT (Cirrus SD-OCT, Carl Zeiss Meditec, Inc, Dublin, CA)[15] uses a super luminescent diode laser to achieve 27,000 A-scans per second. The optic disc cube extends over an area of 6 x 6 mm^2^ covering the optic disc and the peripapillary retina. A built-in algorithm automatically detects the optic disc center and position a calculation circle of diameter 3.46mm (256 A-scans) around the optic disc on the RNFL thickness map.[16] The superior and inferior quadrants are defined as between 45–135 degrees and 225–315 degrees respectively.

**Fig 1:**
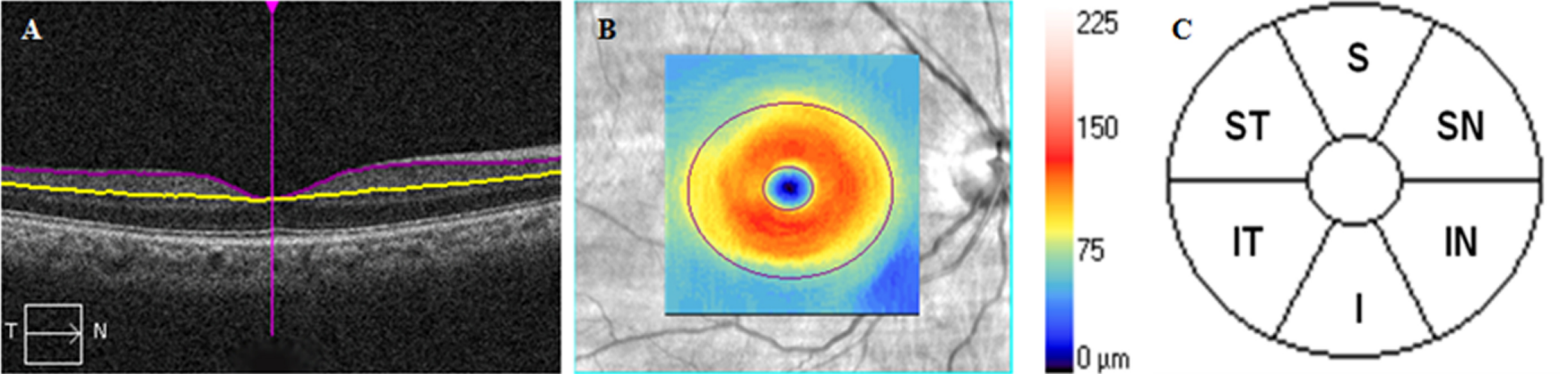
Cirrus SD-OCT images of the macula of the right eye: (A) colour-coded topographic map, (B) separation of macula into 6 sectors∞ and (C) horizontal scan of the macula showing a segmented GCIPL (measured between the purple and yellow horizontal lines).

Cirrus SD-OCT was also used to acquire macular scan using the macular cube 200 X 200 scan protocol. A detailed description of the Ganglion Cell Analysis (GCA) scanning has been reported elsewhere.[17, 18] In brief, the GCA algorithm (Cirrus SD-OCT software version 6) measured the thickness of macular GCIPL inside a 14.13 mm^2^ ellipsoid area with the fovea at the center (**Fig 1A**). The deeper boundary of the RNFL and deeper boundary of the IPL at the macular region were automatically delineated by the GCA algorithm. This isolated segmented layer thus led to measurement of the GCIPL thickness (**Fig 1B**). The average, minimum and 6 sectoral (supero-temporal, superior, supero-nasal, infero-nasal, inferior, infero-temporal) GCIPL thicknesses were measured from the ellipsoid zone centred on the fovea **(Fig 1C).** The minimum GCIPL thickness was defined as the lowest GCIPL thickness over a single meridian crossing the annulus. Only good quality images (signal strength equal to or more than 7) were included in the current analysis.

Confocal scanning laser ophthalmoscopy (CSLO) was performed with the Heidelberg Retinal Tomograph 3 (HRT-3; Heidelberg Engineering, Heidelberg Germany) for imaging of ONH and RNFL. The instrument and its principles are described in details elsewhere.[19] HRT-3 cylindrical lenses were adapted for subjects with astigmatism greater than or equal to 1.0 D. Each image was coupled with a standard deviation (SD) to reflect image quality; a SD higher than 40μm was used as exclusion criterion. The optic disc margin was manually drawn by a single trained operator, which was defined as the inner edge of Elschnig’s ring. The HRT-3 software derived optic disc parameters (e.g. neuroretinal rim area, cup area, rim-to-disc area ratio and cup-to-disc area ratio) automatically using a standard reference plane which was defined at 50 μm posterior to the average retinal height between 350° and 356° along the contour line. For this study, the superior quadrant of the retina comprised of the temporal-superior and nasal-superior sectors i.e. +45 to +135 degrees). The inferior quadrant comprised of the temporal-inferior and nasal-inferior sectors i.e. -135 to -45 degrees).

### Statistical analysis

Statistical analysis was performed using Stata 12.1 (StataCorp LP, College Station, TX). Eye-specific data was used in this analysis. Independent t-test and chi square test were performed to compare the demographics and ocular characteristics of participants with and without glaucoma. Logit model was first performed where the outcome (glaucoma) was regressed on each above-mentioned imaging parameter, while adjusted for age and gender. Predicted probabilities of these respective logit models (for each imaging parameter) were then estimated to produce receiver operating curves, and in turn, area under receiver operating curves (AUC). The method described by Janes and Pepe was then used for the comparisons of AUCs across different imaging parameters.[20] The sensitivity level of each imaging parameter was also calculated for a fixed specificity level of 85%. Generalized estimating equation models with exchangeable correlation structures were applied to account for the correlation between pairs of eyes for each individual in these analyses. A significance level of P < 0.05 was taken for statistical significance.

## Results

Of the original 3,353 study subjects, 1,291 had both HRT and Cirrus SD-OCT scans taken. Among them, 187 had poor HRT scan quality, and another 43 had poor signal strength or motion artefacts in Cirrus SD-OCT scans, thus leaving 1,061 subjects (1,795 eyes) with acceptable quality scans in both HRT and Cirrus SD-OCT included for the final analysis.

**Table 1**compared the demographics and ocular characteristics between participants with (n = 60) and without glaucoma (n = 1,001. The participants with glaucoma were significantly older, more likely to be male, had higher IOP and higher cup-disc ratio. For visual field tests of glaucoma participants, the average mean deviation was -8.95 ± 6.85 dB (median = -7.21 dB; range, -30.36 to 4.86 dB) which places the glaucoma severity in the moderate stage.[21]

**Table 1:** Comparison of demographics and ocular characteristics between non-glaucoma and glaucoma subjects.

	Non-glaucoma (N = 1001, 1709 eyes)	Glaucoma (N = 60, 86 eyes)	P value
Age (years)	61.0 (9.5)	67.5 (9.9)	<0.001
Gender, Male (%)	536 (53.5%)	39 (65%)	0.014
Intra-ocular pressure (mmHg)	14.2 (2.9)	15.4 (3.7)	0.008
Spherical equivalent refractive error (dioptres)	0.67 (2.77)	0.69 (1.95)	0.958
Axial length (mm)	23.9 (1.48)	23.9 (1.21)	0.851
Clinical vertical cup-to-disc ratio	0.39 (0.12)	0.69 (0.15)	<0.001
Central corneal thickness (um)	552.9 (33.8)	539.9 (31.8)	0.019
Best-correctable visual acuity (LogMar)	0.05 (0.09)	0.10 (0.09)	0.001
Mean deviation of visual fields (dB)	-1.32 ± 2.56	-8.95 ± 6.85	<0.001
Vertical cup-to-disc ratio (HRT-3)	0.37 (0.22)	0.62 (0.21)	<0.001
Vertical cup-to-disc ratio (Cirrus SD-OCT ONH)	0.50 (0.14)	0.72 (0.09)	<0.001
Mean RNFL thickness (HRT-3) (µm)	263.3 (78.1)	188.4 (72.7)	<0.001
Mean RNFL thickness (Cirrus SD-OCT ONH) (µm)	96.5 (9.8)	75.4 (14.6)	<0.001
Mean macular GCIPL thickness (µm)	82.6 (6.3)	72.8 (9.3)	<0.001
Minimum macular GCIPL thickness (µm)	79.6 (7.5)	66.3 (12.0)	<0.001

All continuous variables are presented in mean (standard deviation)

SD-OCT: Spectral-domain Optical Coherence Tomography; ONH: optic nerve head; HRT: Heidelberg Retinal Tomography; GCIPL: ganglioan cell-inner plexiform layer

**Table 2**compared the diagnostic performance of each measurement from Cirrus macular GCIPL, Cirrus ONH and HRT-3 scans. The best performing measurements for Cirrus macular GCIPL imaging, Cirrus ONH and HRT-3 were minimum GCIPL thickness (AUC = 0.89, 95% CI 0.83 to 0.95), vertical CDR (AUC = 0.94, 95% CI 0.91 to 0.98) and vertical cup-disc ratio (CDR) (AUC = 0.86, 95% CI 0.81 to 0.92) respectively. Post-hoc analysis comparing the best parameter of each imaging tool showed that vertical CDR measured by Cirrus ONH scan performed better than vertical CDR measured by HRT-3 (P = 0.004). However, the difference between vertical CDR measured by Cirrus ONH scan was not significantly different from minimum GCIPL thickness (P = 0.066). In addition, inferior RNFL thickness (AUC = 0.84, 95% CI 0.91 to 0.97) measured using Cirrus OCT showed very similar diagnostic performance as Cirrus OCT’s VCDR and was also better when compared to other parameters measured by HRT-3 and Cirrus SD-OCT macular GCIPL scan (all P≤0.007). **Fig 2**compared the AUC of the best diagnostic parameters from all three imaging modalities.

**Fig 2:**
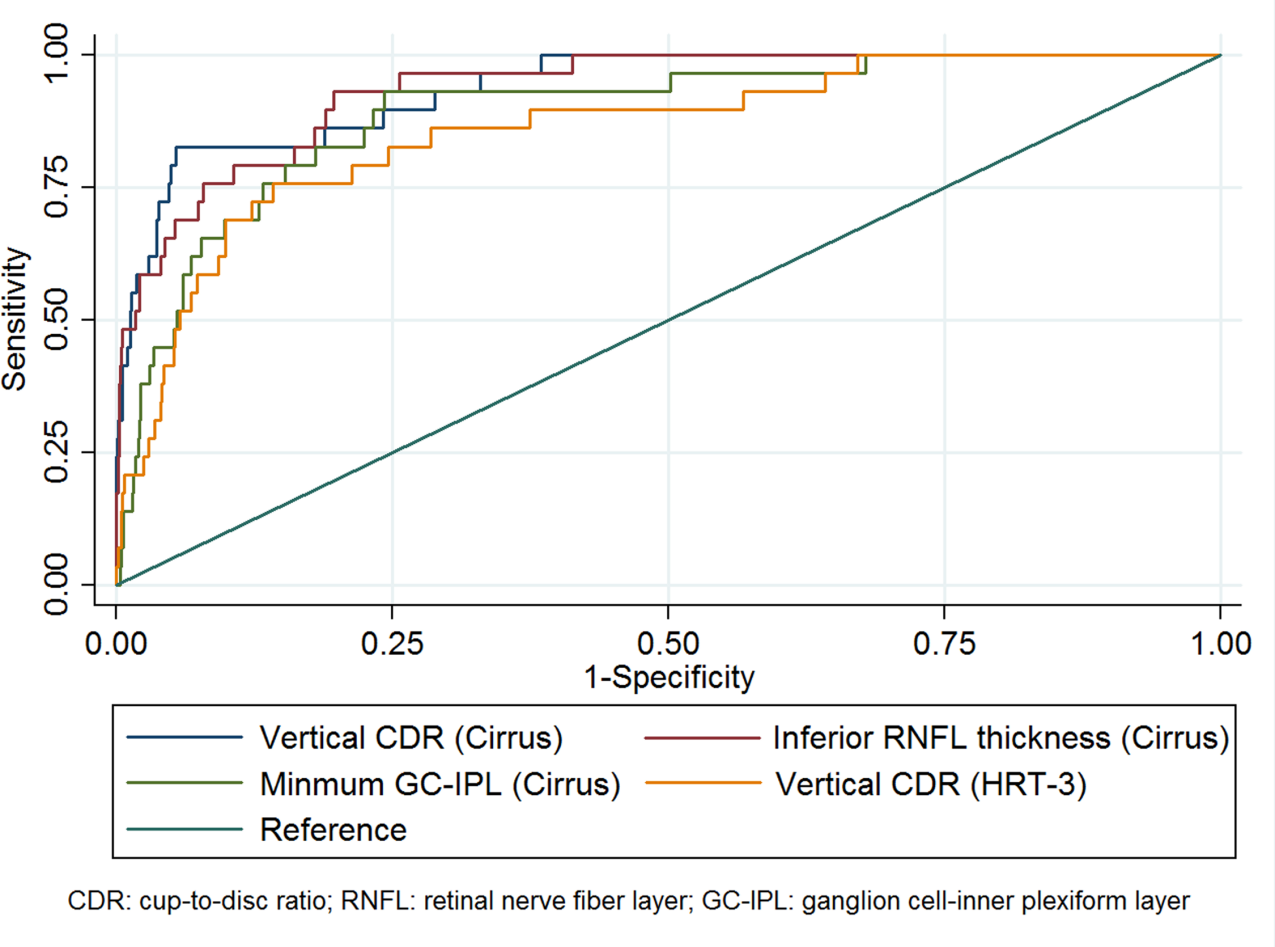
Comparison of area under receiver operating characteristic curve between the best parameters of Heidelberg Retinal Tomography -3 (HRT-3), Cirrus Spectral Domain-optical coherence tomography (SD-OCT) optic nerve head analysis and Cirrus SD-OCT macular ganglion cell-inner plexiform layer (GCIPL) analysis.

**Table 2:** Comparison of the area under the receiver-operating curves between the parameters of cirrus SD-OCT macular ganglion cell-inner plexiform layer analysis and optic nerve head analysis, and Heidelberg retinal tomography-3 (HRT-3).

	**Cirrus SD-OCT macular GCIPL**		**Cirrus SD-OCT ONH and RNFL**		**HRT-3**
**Parameters**	**AUC (95% CI)**	**Parameters**	**AUC (95% CI)**	**Parameters**	**AUC (95% CI)**
Minimum GCIPL thickness	0.89 (0.83 to 0.95)	Vertical CDR	0.94 (0.91 to 0.98)	Vertical CDR	0.86 (0.81 to 0.92)
Inferior GCIPL thickness	0.87 (0.80 to 0.95)	Inferior RNFL thickness	0.94 (0.91 to 0.97)	Rim-to-disc area ratio	0.85 (0.80 to 0.92)
Average GCIPL thickness	0.87 (0.81 to 0.94)	Average CDR	0.91 (0.87 to 0.95)	Superior Rim area	0.82 (0.74 to 0.89)
Superior GCIPL thickness	0.85 (0.78 to 0.93)	Average RNFL thickness	0.90 (0.86 to 0.95)	Mean RNFL Thickness	0.81 (0.75 to 0.89)
-	-	Superior RNFL thickness	0.90 (0.85 to 0.95)	Average Rim area	0.78 (0.69 to 0.86)
-	-	-	-	Inferior rim area	0.78 (0.69 to 0.87)

AUC: area under the receiver-operating curve; RNFL: retinal nerve fiber layer, CDR: cup-to-disc ratio; ONH: optic nerve head; GCIPL: ganglion cell-inner plexiform layer

**Table 3**compared the sensitivity of selected parameters from all three imaging modalities at a specificity level of 85%. Macular minimum GCIPL thickness showed sensitivity of 60.53% (95% CI 46.4 to 73.0) but vertical CDR measured using Cirrus OCT ONH scan showed the highest sensitivity (88.64%, 95% CI 75.4 to 96.2) followed by inferior RNFL thickness (81.81%, 95% CI 67.3 to 91.8). Comparing the sensitivity of the best parameters of each imaging tool, vertical CDR measured by Cirrus SD-OCT ONH scan performed better than vertical CDR measured by HRT-3 (65.3%, P = 0.003); and minimum GCIPL thickness (60.5%, p<0.001). Other parameters from HRT-3 and Cirrus SD-OCT generally showed moderate to poor sensitivity in detecting glaucoma at a fixed specificity level of 85%.

**Table 3:** Comparison of sensitivity values (at 85% specificity) between the parameters of cirrus SD-OCT macular ganglion cell-inner plexiform layer analysis and optic nerve head analysis, and Heidelberg retinal tomography-3 (HRT-3).

	Cirrus SD-OCT macular GCIPL		Cirrus SD-OCT ONH and RNFL		HRT-3
Parameters	Sensitivity (95% CI)	Parameters	Sensitivity (95% CI)	Parameters	Sensitivity (95% CI)
Minimum GCIPL thickness	60.53 (46.4 to 73.0)	Vertical CDR	88.64 (75.4 to 96.2)	Vertical CDR	65.3 (60.3 to 70.0)
Inferior GCIPL thickness	57.89 (43.3 to 71.2)	Inferior RNFL thickness	81.82 (67.3 to 91.8)	Rim- to-disc area ratio	65.3 (60.3 to 70.0)
Average GCIPL thickness	52.63 (38.7 to 66)	Average CDR	77.27 (62.2 to 88.5)	Superior rim area	44.47 (39.5 to 49.6)
Superior GCIPL thickness	47.37 (34.2.0 to 612)	Average RNFL thickness	70.45 (54.8 to 83.2)	Average rim area	42.16 (37.2 to 47.2)
-	-	Superior RNFL thickness	70.45 (54.8 to 83.2)	Inferior rim area	41.65 (36.7 to 46.7)
-	-	-	-	Mean RNFL Thickness	41.39 (36.4 to 46.5)

ONH: optic nerve head; GCIPL: ganglion cell-inner plexiform layer; RNFL: retinal nerve fiber layer, CDR: cup-disc ratio.

## Discussion

Structural imaging tools are an important aspect of diagnostics and monitoring in the glaucoma clinics. However, the diagnostic performances of glaucoma imaging tools are inconsistent in clinic settings and population-based studies, with results more favourable in the former.[22–24] These could be attributed to the differences in glaucoma severity which tend to be more severe and symptomatic in patients attending the glaucoma clinic. There is also a selection bias towards a glaucomatous-looking ONH detected in a clinic which subsequently results in a structural imaging test being more likely to be abnormal. In theory, imaging tools are ideal for screening purpose as they are quick, non-contact, operated by a skilled technician, objective and highly-reproducible. However, there are also limitations which adversely affect the diagnostic accuracy of these imaging tools which include media opacity affecting the image quality,[25] segmentation errors,[26] myopic optic disc[27] and presence of peripapillary atrophy.[28]

For OCT to be used as a glaucoma a screening tool in a population setting, we have to consider the relative low prevalence of glaucoma. In this regard, and it is reasonable for an imaging tool to be judged based on its sensitivity at a higher fixed specificity level of 85%, which was adopted in our study. This provides a generally acceptable balance between a good yield and cost-effectiveness for a screening tool. At a fixed specificity of 85%, the best parameter of Cirrus SD-OCT has a sensitivity of 88.64% (95% CI 75.4, 96.2) which means that only missing one-tenth of glaucoma cases if Cirrus SD-OCT is used for glaucoma screening in population setting. In contrast, at fixed specificity of 85%, inferior or minimum macular GCIPL thickness measurment will miss up to 40% of glaucoma eyes, which seem to suggest that macular GCIPL scan might have limited value for glaucoma screening in an Asian population. Similarly, the best HRT-3 parameter (vertical CDR) will also miss up to 35% of glaucoma eyes in a population screening setting. It is not surprising that vertical CDR measured by Cirrus SD-OCT and HRT-3 differed in their diagnostic performance as the measured vertical CDR has been shown to be poorly correlated.[29] This is primarily due to differences in how the vertical CDR is derived. In HRT-3, retinal surface landmarks, which are not anatomically consistent,[30] are used to identify the disc edges compared to Cirrus SD-OCT which used the termination of Bruch's membrane to delineate the optic disc edge.[31] The latter is considered a more accurate representation of the disc diameter which also forms the basis of a newer ONH parameter, Bruch’s membrane opening–minimum rim width.[32] In addition, in HRT-3, an observer arbitrarily identifies the disc margin which adds to inter- and intra-observer variability compared to Cirrus SD-OCT which automatically delineates the disc margin.

Macular GCIPL thickness measurement is a relatively newer glaucoma detection tool which is postulated to be involved in early glaucoma. Shoji et al. also suggested that GCIPL thickness measurement are less likely to be influenced by changes in axial length or refractive errors which is an advantage in an Asian population with a high prevalence of myopia.[33] Our study is the first to show good diagnostic performance of macular GCIPL thickness measurement for glaucoma screening in an Asian population. Both minimal and inferior GCIPL thickness demonstrated good AUC of above 0.80 which is consistent with previous studies.[34, 35] The inferior macula ganglion cell layer was also postulated to be the earliest and most affected by glaucomatous changes[36] and our findings were consistent with other studies.[34] Although our study showed that macular GCIPL thickness had good diagnostic accuracy for glaucoma, it was still significantly less sensitive than Cirrus SD-OCT ONH and RNFL scan in a population-based setting. This is in contrast to clinic-based case-control studies which reported a better or at least comparable performance between macular GCIPL measurements and peripapillary ONH parameters.[12, 35] Similar to our study, in the population-based Rotterdam study, Springelkamp et al. reported a high AUC of 0.93 but a relatively poorer sensitivity of inferior macular GCIPL thickness which translated into underdiagnosing close to one-third of glaucomatous eyes in a population-based screening (based on abnormal VF loss and ONH changes).Compared to our study, the slight difference in diagnostic performances can be attributed to the differences in population demographics, different OCT machines used and definition of ganglion cell layer thickness. Co-existing macular pathology such as diabetic maculopathy, age-related macular degeneration and myopia[27] may also play a role in influencing the diagnostic performance of GCIPL scans although we have already excluded eyes with these conditions in our study. The role of macular GCIPL imaging for glaucoma could possibly be beneficial when used in combination with RNFL thickness for detection of early glaucoma.[37]

Our study has several strengths which include a standardized ocular examination and glaucoma imaging and a large population-based study comprising all 3 imaging. The definition of glaucoma used in our study was also used in established population studies which include structural (glaucomatous optic neuropathy) and/or functional (abnormal visual fields) criteria.[35, 38] Such definition is better than other studies which defined glaucoma based on visual field tests only, the latter definition could underestimate and unnecessarily reduce the number of eyes classified as glaucoma in a population study by excluding pre-perimetric glaucoma. In addition, as structural ONH changes could precede visual field changes, the latter definition could only include glaucoma which are more advanced in severity which is not an ideal representation of glaucoma severity in a population screening setting. A possible limitation for using structural definition for glaucoma in our study may result in bias which favours imaging tools especially stereometric parameters based on the ONH measurements. However, there are significant differences and poor agreement between clinical assessment of cup-to-disc ratio and structural imaging modalities and even between HRT-3 and SD-OCT measurements.[29, 39] The HRT tend to underestimate vertical CDR by as much as between 0.10 and 0.24[40] and OCT tends to overestimate vertical CDR by as much as between 0.08 and 0.11.[29, 41]

In conclusion, the diagnostic performance of macular GCIPL scan is inferior compared to vertical CDR measured by Cirrus OCT ONH scan in glaucoma screening for an Asian population. SD-OCT ONH scan performed the best in detecting glaucoma with a sensitivity of 88.64% at 85% specificity using vertical CDR of the ONH. Both SD-OCT macular GCIPL and HRT-3 ONH measurements may miss up to 40% of eyes with glaucoma and may not be suitable as screening imaging tools for glaucoma in an Asian population.
